# Monitoring of efficacy, tolerability and safety of artemether–lumefantrine and artesunate–amodiaquine for the treatment of uncomplicated *Plasmodium falciparum* malaria in Lambaréné, Gabon: an open-label clinical trial

**DOI:** 10.1186/s12936-019-3015-4

**Published:** 2019-12-16

**Authors:** Bayode R. Adegbite, Jean R. Edoa, Yabo J. Honkpehedji, Frejus J. Zinsou, Jean C. Dejon-Agobe, Mirabeau Mbong-Ngwese, Fabrice Lotola-Mougueni, Erik Koehne, Albert Lalremruata, Andrea Kreidenweiss, The T. Nguyen, Jutta Kun, Selidji T. Agnandji, Bertrand Lell, Abdou R. Safiou, Fridia A. Obone Atome, Ghyslain Mombo-Ngoma, Michael Ramharter, Thirumalaisamy P. Velavan, Benjamin Mordmüller, Peter G. Kremsner, Ayola A. Adegnika

**Affiliations:** 1 Centre de Recherches Médicales de Lambaréné, BP: 242, Lambaréné, Gabon; 2 Institut für Tropenmedizin, Universität Tübingen, Tübingen, Germany; 3 Center of Tropical Medicine and Travel Medicine, Department of Infectious Diseases, Amsterdam University Medical Centers, University of Amsterdam, Amsterdam, The Netherlands; 4 Department of Parasitology, Leiden University Medical Center, Leiden, The Netherlands; 5 Programme National de Lutte contre le paludisme, Libreville, Gabon; 6 Hôpital Albert Schweitzer de Lambaréné, Lambaréné, Gabon; 7 Vietnamese-German Center for Medical Research, Hanoi, Vietnam; 8 Division of Infectious Diseases and Tropical Medicine, Department of Medicine 1, Medical University of Vienna, Vienna, Austria; 9 Department of Tropical Medicine, Bernhard Nocht Institute for Tropical Medicine, Hamburg, Germany; 10 Department of Medicine, University Medical Center Hamburg-Eppendorf, Hamburg, Germany; 11 German Center for Infection Research, Tübingen, Germany

**Keywords:** Malaria, Artemether–lumefantrine, Artesunate–amodiaquine, Efficacy, Tolerability, Safety, Lambaréné, Gabon

## Abstract

**Background:**

Malaria remains a major public health problem, affecting mainly low-and middle-income countries. The management of this parasitic disease is challenged by ever increasing drug resistance. This study, investigated the therapeutic efficacy, tolerability and safety of artemether–lumefantrine (AL) and artesunate–amodiaquine (AS–AQ), used as first-line drugs to treat uncomplicated malaria in Lambaréné, Gabon.

**Methods:**

A non-randomized clinical trial was conducted between October 2017 and March 2018 to assess safety, clinical and parasitological efficacy of fixed-doses of AL and AS–AQ administered to treat uncomplicated *Plasmodium falciparum* malaria in children aged from 6 months to 12 years. After 50 children were treated with AL, another 50 children received ASAQ. The 2009 World Health Organization protocol for monitoring of the efficacy of anti‑malarial drugs was followed. Molecular markers *msp1* and *msp2* were used to differentiate recrudescence and reinfection. For the investigation of artemisinin resistant markers, gene mutations in P*fk*13 were screened.

**Results:**

Per-protocol analysis on day 28 showed a PCR corrected cure rate of 97% (95% CI 86–100) and 95% (95% CI 84–99) for AL and AS–AQ, respectively. The most frequent adverse event in both groups was asthenia. No mutations in the *kelch*-*13* gene associated with artemisinin resistance were identified. All participants had completed microscopic parasite clearance by day 3 post-treatment.

**Conclusion:**

This study showed that AL and AS–AQ remain efficacious, well-tolerated, and are safe to treat uncomplicated malaria in children from Lambaréné. However, a regular monitoring of efficacy and a study of molecular markers of drug resistance to artemisinin in field isolates is essential.

*Trial registration* ANZCTR, ACTRN12616001600437*. Registered* 18 November, http://www.anzctr.org.au/TrialSearch.aspx?searchTxt=ACTRN12616001600437p&isBasic=True

## Background

The World Health Organization (WHO) reported 219 million cases of malaria and 435,000 deaths due to malaria in 2017 [1]. A total of 35,244 cases of malaria were notified in Gabon in 2017 [2]. Since 2001, the WHO has recommended artemisinin-based combination therapy (ACT) for uncomplicated malaria treatment [3]. However, resistance to artemisinin has been detected in Cambodia, Lao People’s Democratic Republic, Myanmar, Thailand and Viet Nam, five countries of the Greater Mekong Sub-region. And it is spreading [4, 5]. The P*fkelch*13 C580Y haplotype of *Plasmodium falciparum* was identified to be associated with resistance to artemisinin derivates [6].

The efficacy of ACT is being monitored in many Africa countries. There have been some reports of delayed parasite clearance during routine therapeutic efficacy studies of ACT. However, these reports have not been consistent over time [7]. Between 2010 and 2016, the overall average efficacy rates of artesunate–amodiaquine and artemether–lumefantrine, which are the first-line treatment policies used in most African countries, were 98.3% and 97.9%, respectively [7]. A study performed between 2007 and 2009 in Lambaréné and Fougamou among children 6–59 months of age, reported a day 28 cure rate of 98.6% and 94.7% for AL and AS–AQ, respectively [8] Since 2013, the National Malaria Control Programme (NMCP) of Gabon has updated the malaria treatment guideline by recommending artesunate–amodiaquine (AS–AQ), and artemether–lumefantrine (AL) as first-line treatment for uncomplicated malaria cases. Two therapeutic efficacy studies assessing these first-line drugs in Gabon were performed in Libreville and Franceville (unpublished data). No recent study was performed to assess AS–AQ and AL efficacy to treat uncomplicated *P. falciparum* malaria in Lambaréné. To ensure that the treatments recommended in the national treatment policy are efficacious, the WHO recommends that malaria-endemic countries perform routine monitoring of anti-malarial drug efficacy at least once every 24 months in order to detect changes in therapeutic efficacy. Furthermore, an increased prevalence of the wild-type allele N86Y of the *P. falciparum* multidrug resistance-1 (*Pfmdr1*) gene was described in Lambaréné and surroundings [9, 10]. The presence of this haplotype in the Lambaréné region is an additional reason to follow the WHO recommendation of a regular monitoring of the resistance of artemisinin derivatives and the therapeutic efficacy in this area. This study was carried out to assess the therapeutic efficacy, tolerability and safety of AL and AS–AQ in Lambaréné using the 2009 WHO protocol for surveillance of anti-malarial drugs [11].

## Methods

### Study site and period

The study was conducted at the Centre de Recherche Médicales de Lambaréné (CERMEL) a former Medical Research Unit in Lambaréné [12], Gabon from October 2017 to March 2018. The CERMEL, is located in Lambaréné a capital city of the Moyen Ogooué Province of Gabon, in the Central African rainforest belt. Lambaréné and surrounding villages are highly endemic for *P. falciparum* malaria [13–15], and show high levels of resistance of *P. falciparum* to chloroquine [16–19] and sulfadoxine–pyrimethamine [20]. The region presents a highly persistent of PfCRT mutant [21]. However, a recent analysis of samples from the region indicated the absence of the *k13* gene mutation [9, 22].

### Study design

The study was an open-label clinical trial with a sequential enrolment of patients treated with AL or AS–AQ. After 50 children were treated with AL, another 50 children received AS–AQ. The primary objective was to assess the PCR corrected cure rates at day 28; the secondary objectives were to assess patterns of fever and parasite clearance; changes in haemoglobin levels after treatment by AL or AS–AQ as well as their safety and tolerability; and investigate the presence of artemisinin resistance molecular markers.

### Study population and recruitment

All children aged between 6 months and 12 years who attended health care facilities in Lambaréné for signs and symptoms of malaria were invited to participate in the study. The inclusion criteria were: temperature ≥ 37.5 °C, or history of fever during the past 24 h; mono-infection with asexual *Plasmodium falciparum*; parasite density between 1000 and 200,000 per µl; haemoglobin level ≥ 8 g/dl and absence of signs and/or symptoms of severe malaria. Participants were allocated in two groups (AL and AS–AQ). The first set of 50 included participants received AL and the second set received AS–AQ.

### Sample size

The required sample size was calculated by using a single population proportion formulae based on the revised WHO protocol [11]. Based on an expected PCR-corrected cure rate of 98% for both AL and AS–AQ in Lambaréné region [8], a 95% confidence level and 5% precision, the initial calculated sample size was 30 for each treatment arm. This number was increased to 50 to account for any lost to follow-up and study withdrawal post recruitment [11].

### Laboratory procedures

Venous blood was collected to assess haemoglobin level as well as to perform malaria thick and thin smear using WHO protocol. For the late, three slides (two thick blood smears and one thin blood smear) were prepared. One was rapidly stained (Giemsa 10% for 15 min) for rapid screening. The other two slides were stained using Giemsa at 3% for 60 min. These two slides were used to determine the baseline parasite density and species identification. For quality control purposes, all malaria slides were read independently by two qualified microscopists, and any discordant readings were re-examined by a third qualified independent microscopist. Discordance was defined as divergences between the first and second microscopists regarding presence/absence of asexual or sexual parasites; species diagnosis; and parasite counts of more than 20% difference was notified. The final parasite density was determined by taking the average of the parasitaemia of the two closest reading. The haematological parameter (haemoglobin level and haematocrit) was determined using haematology analyser (ABX Pentra 60, Horiba). Filter paper (Whatman) blood spots were obtained on day 0 and on each day where thick blood smears were performed for polymerase chain reaction (PCR) genotyping. Polymerase chain reaction analysis was done to distinguish recrudescence from new infection on sample with treatment failure. Blood spotted and dried on filter paper were used to extract nucleic acids by using mini spin-column (QIAamp Blood DNA mini kit, Qiagen) based on the kit manual. Purified nucleic acids were eluted using 50 µL elution buffer and stored at − 20 °C for further use. Genotyping of *Plasmodium falciparum* parasite was performed with blood collected at pre-treatment and at the time of recurrent malaria after treatment. In brief, a gene coding for *Plasmodium falciparum* merozoite surface protein 1 and 2 (*Pfmsp1*, *Pfmsp2*) were amplified by a nested-PCR method. Allelic family-specific primers based on the WHO recommendation for genotyping were used and listed in Additional file 1: Tables S1, S2 with detail PCR conditions. The amplified products were analysed on the QIAxcel advanced system using the DNA high-resolution kit (Qiagen, Hilden, Germany). The band sizes were calculated with the QIAxcel Screen Gel software and the height cut off for minority clones was set at 10% of the dominant peak. The *Pfk13* markers of artemisinin resistance was evaluated on sample of participants with treatment failure. For the investigation of artemisinin resistant markers, the following gene mutations: M476I, Y493H, R539T, I543T and C580Y in the P*fk*13 were screened using the primer pairs indicated by Ariey et al. [23]. In brief, 10 ng of parasite genomic DNA were added to a 20 µL reaction mixture containing 1× PCR buffer (20 mM Tris–HCl pH 8.4, 50 mM KCl, 2.5 mM of MgCl_2_), 0.125 mM of dNTPs, 0.25 mM of each primer and 1U Taq DNA polymerase (Qiagen, Hilden, Germany). The PCR reaction was run on a PTC-200 Thermal cycler (MJ Research, Waltham, USA). PCR products were visualized through electrophoresis on a 1.2% agarose gel stained with SYBR green I in 1× Tris-electrophoresis buffer (90 mM Tris–acetate, pH 8.0, 90 mM boric acid, 2.5 mM EDTA). Subsequently, PCR products were purified (Exo-SAP-IT, USB, Affymetrix, Santa Clara, CA, USA) and directly used as templates for DNA sequencing using the BigDye terminator v. 1.1 cycle sequencing kit (Applied Biosystems, Foster City, USA) on an ABI 3130XL DNA sequencer. Polymorphisms were identified by assembling the sequences with the reference sequence of the Pfk13 (NC_004331.2) genes using the Codon code Aligner 4.0 software [24] and visually reconfirmed from their electropherograms.

### Participants treatment and follow-up

The fixed-dose combinations of prequalified anti-malarial drugs were supplied by the Gabon’s WHO headquarters. The drugs were administered as instructed by the manufacturer, for 3 days based on body weight. Children in AL group received weight-based 20/120 mg of artemether–lumefantrine from, Ipca, Laboratories, India (Batch number DY2036341, expiring 10/2018). The schedule of treatment was six doses distributed as following H0, H8, H24, H36, H48, and H60. Children weighing 5 to < 15 kg were given one tablet per dose; those weighing 15 to < 25 kg were given two tablets per dose; those weighing 25 to < 35 kg were given three tablets per dose; and those weighing 35 kg and more were given four tablets per dose. The first dose of AL was given under a direct observation of the investigator, while the parents outside of investigator’s supervision gave the second dose 8 h following the initial administration. Two doses daily on the 2nd and 3rd days were given under direct observation of study team. Children in AS–AQ group received single daily weight-based products from Winthrop^®^, Sanofi-Aventis, France. The products were 25/67.5 mg (Batch number 5MA082, expiring 09/2018) for children weighing 5 to < 9 kg; 50/135 mg (Batch number 6MA095, expiring 12/2018) for children weighing 9 to < 18 kg; 100/270 mg (Batch number 5MA392, expiring 09/2018) for children weighing 18 to < 36 kg and children weighting 36 and more received two tablet of AS–AQ 100/270 mg. The participant was observed for 30 min after dosing to ascertain that drugs were not rejected. Children who vomited during the observation period were re-treated with the same dose of anti-malarial and observed for an additional 30 min. Children with repeated vomiting were given parenteral therapy with artesunate and excluded from the study. Children who showed signs/symptoms of severe malaria were withdrawn from the study. The participants enrolled were followed-up for up to 28 days. They were visited on days 1, 2, 3, 4, 7, 14, 21, and 28 (day of first drug administration was counted as day 0). At each follow-up visit, clinical information and possible solicited and unsolicited adverse events were collected on a case report form. Thick blood smears were performed on days 2, 3, 7, 14, 21, and 28 and any day within the follow-up period that a participant came with suspected signs and symptoms of malaria.

Participants were withdrawn from the study during the follow-up period if they met one of the following criteria: detection of non-falciparum mixed or mono-infection, medication with any anti-malarial drug and consent withdrawal by the parents or legal guardian. If the participant missed a visit day he was classified as lost to follow-up (1 day early or delay was accepted if it occurred between days 7 to 28 of follow-up). Participants treatment outcome were categorized as adequate clinical and parasitological response (ACPR), early treatment failure (ETF), late clinical treatment failure (LCF), late parasitological treatment failure (LPF).

### Safety

The safety and tolerability of the drugs were assessed at each scheduled visit. Any adverse event that occurred during the follow-up was recorded in the case report form; mentioning the nature, the severity, the start date, the end date, the treatment administered to treat it and the probable association with the investigational drugs. Participants were advised to come to the study site anytime they have a new symptom during the follow-up.

### Data management

WHO Standardized Microsoft Excel data collection sheet were used to enter demographic, clinical and parasitological information for each participant [25]. Data were independently entered by two persons and reviewed by a third person. Any difference between these two data entered was automatically notified by the Excel software and was reviewed and corrected by a third person after checking the information on the case report form.

### Statistical analysis

The efficacy of the treatment was analysed using two methods according to WHO recommendation [11]. The Kaplan–Meier survival analysis calculated the PCR-adjusted cumulative incidence of success up to day 28. Participants who were lost to follow-up, or withdrawn, were censored at the last visit day. Participant who had re-infection were also censored at the last day where the microscopy was negative. Participants with indeterminate PCR results were excluded from the analysis. The second analysis method was per-protocol. Here, the efficacy rate was the proportion of ACPR among the patients for whom the therapeutic endpoint was reached. The pattern of fever and parasite clearance were assessed using respectively the proportion of children with temperature ≥ 37.5 °C or history of fever reported by the parent and the proportion of children with positive parasitaemia within the 1st week of follow-up. The haematological response was assessed by comparing pre-treatment and post treatment mean haemoglobin level (day 0 and day 28 or any available post-treatment assessment). The qualitative variables were described as proportions with a 95% confidence interval, and the quantitative variables were described as mean and standard deviation (SD) if normally distributed, or by the median and interquartile range (IQR) or geometric mean if log transformed. Student’s t-test or Wilcox-test were used to compare quantitative variables. Proportions were compared using Chi square and Fisher’s exact t-test. Differences were considered significant at p < 0.05. All analyses were performed using R software (version 3.5.1).

## Results

### Participants’ baseline characteristics and dispositions

**Fig. 1:**
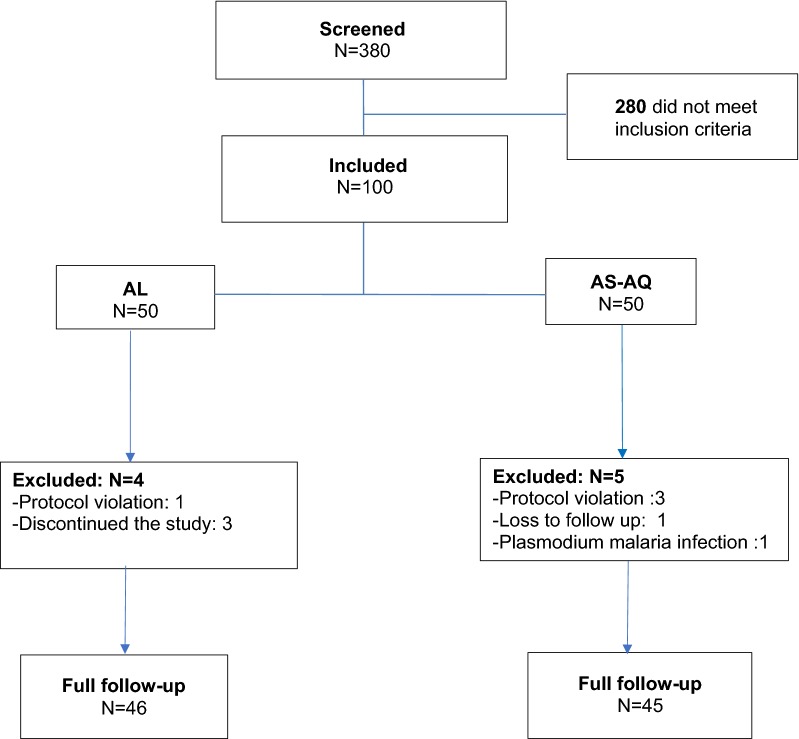
Details on study inclusion and follow up progress

A total of 380 children who reported fever or history of fever were screened in this study. A total of 115 had positive thick blood smear, of whom 100 were eligible (Fig. 1). Median age was 8 [IQR: 5–10] years in AL group and 9 [IQR: 7–10] years in AS–AQ group. The male/female ratio were 1.5 for AL and 0.79 for AS–AQ groups. The geometric mean of asexual parasites density was 13,772 parasites/µL (95% CI 9637–19,680) and 17,648 parasites/µL (95% CI 12,010–23,059) for AL and AS–AQ groups, respectively. There is no evidence of difference in the baseline characteristics of the two groups allocations (Table 1). In AL group, three participants discontinued the study and one was withdrawn due to protocol violation. Five participants in AS–AQ group were withdrawn due to: loss to follow-up (1), protocol violation (3) and infection to *Plasmodium malariae* during the follow-up (Fig. 1).

**Table 1:** Baseline characteristics of participants

Characteristic	AL	AS–AQ
Sex ratio (M/F)	1.5 (30/20)	0.79 (22/28)
Age median [IQR] year	8 [5–10]	9 [7–10]
Mean temperature [SD] °C	38.6 [1.0]	38.1 [1.2]
Weight mean [SD] kg	22.7 [8.3]	26 [7.9]
Haemoglobin mean [SD] g/dL	10.2 [1.4]	10.4 [1.5]
Asexual parasitaemia geometric mean [95% CI]/µL	13,772 [9637–19,680]	17,648 [12,010–23,059]

*SD* standard deviation, *IQR* interquartile range

### Primary outcomes

**Fig. 2:**
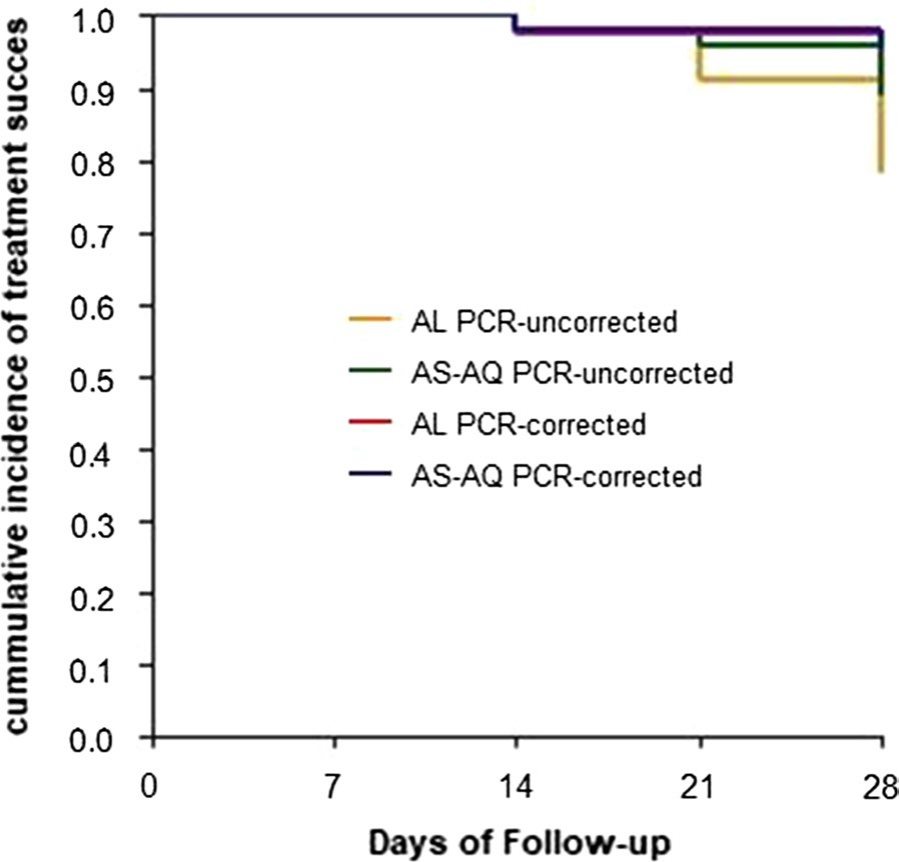
Kaplan–Meier curves showing treatment success cumulative proportion up to day 28 of follow-up PCR uncorrected and PCR-corrected for AL and AS–AQ

A 28-day follow-up was achieved for 46 and 45 participants who received AL and AS–AQ respectively. The per-protocol (PP) analysis with PCR uncorrected outcomes showed a day 28 cure rate of 78%, (95% CI 64–89) for AL and 89% (95% CI 76–96) for AS–AQ. No early treatment failure was observed following the treatment with both drugs. PCR genotyping analysis of 15 participants who had reappearance of malaria parasites, 3 (1 for AL and 2 for AS–AQ) were confirmed as recrudescence while the others were new infections. Two PCR reactions in AL group samples resulted in no amplification; most likely due to insufficient blood in the filter paper. These participants were excluded from analysis [25]. The PCR corrected, PP cure rate was 97% (95% CI 86–100) for AL and 95 (95% CI 84–99) for AS–AQ (Table 2). The PCR-corrected Kaplan–Meier survival analyses for AL and AS–AQ showed similar evolution (Fig. 2). The cumulative cure rate on days 28 was 0.96 and 0.98, respectively.

**Table 2:** Per protocol outcome of treatment with AL and AS–AQ

Outcome	AL			AS–AQ		
	n	%	[95% CI]	N	%	[95% CI]
PCR-uncorrected						
ETF	0	0	[0–8]	0	0	[0–8]
LCF	7	15	[6–29]	2	4	[1–15]
LPF	3	7	[1–18]	3	7	[1–18]
ACPR	36	78	[64–89]	40	89	[76–96]
ETF	0	0	[0–10]	0	0	[0–8]
PCR-corrected						
LCF	0	0	[0–10]	1	2	[0–13]
LPF	1	3	[0–14]	1	2	[0–13]
ACPR	36	97	[86–100]	40	95	[84–99]

*ACPR* adequate clinical and parasitological response, *ETF* early treatment failure, *LCF* late clinical treatment failure, *LPF* late parasitological treatment failure

### Secondary outcomes

**Fig. 3:**
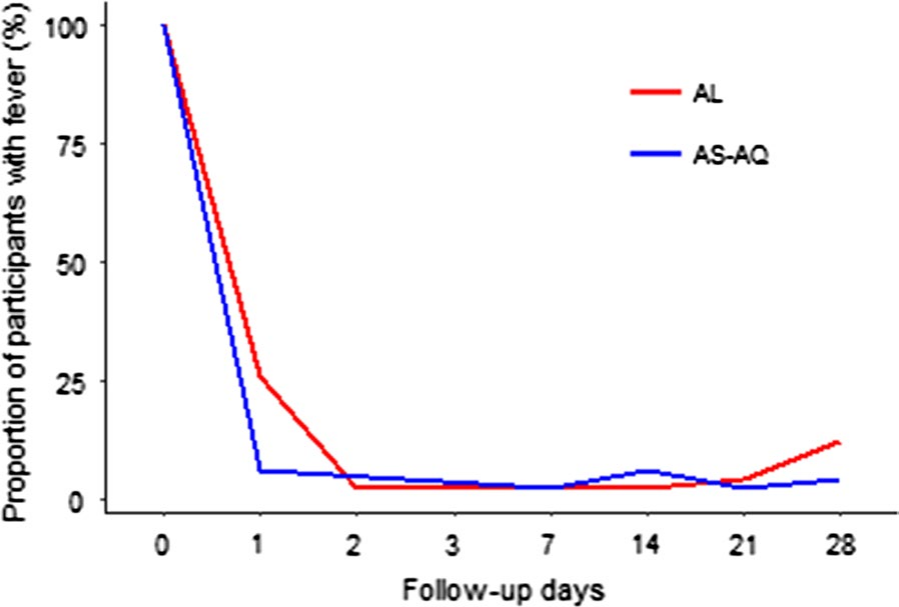
Proportion of participants with fever during the follow-up

The prevalence of fever decreased by 73% for AL and 94% for AS–AQ within 24 h of the follow-up (Fig. 3). Only one participant was febrile in AS–AQ group on day 3. However, all participants were afebrile on day 7. Febrile participants after day 7 were those who had reinfection or treatment failure. The parasite clearance on day 2 was 95% for AL and 98% for AS–AQ; and no participant was parasitaemic between day 3 and day 14 The *Pfk13* variants M476I, Y493H, R539T, I543T, and C580Y reported to occur in Southeast Asia were not observed in the study population. The haemoglobin concentration significantly increased from 10.4 g/dL (SD 1.5) at baseline to 11.1 g/dL (SD 1.8) in AS–AQ group on day 28 post-treatment (P = 0.02). Within the AL group the haemoglobin concentration (SD) increased from 10.2 (1.4) at baseline to 10.8 (2.0), however this increase was not statistically significant (P = 0.82).

### Safety, tolerability, and serious adverse events

The number of participants with at least one adverse event during the study period was 7/50 (14%) for AL and 17/50 (34%) for AS–AQ. The most common adverse events in decreasing order were asthenia, loss of appetite, vomiting, and diarrhoea (Table 3). Three serious adverse events (hospitalization due to pneumonia, severe anaemia, and rhinotracheitis) were registered during the follow up in AS–AQ group, but none was related to the study drug. They were hospitalised and managed at paediatric ward successfully.

**Table 3:** Adverse events during the follow-up period

Adverse event	AL	AS–AQ
Asthenia	4	16
Loss of appetite	2	2
Diarrhoea	2	0
Vomiting	1	2
Nausea	0	1
Dizziness	0	1
Hospitalization^a^	0	3

^a^Pneumonia, severe anaemia, and rhinotracheitis

## Discussion

This study reports a prospective assessment of two first line anti-malarial drugs recommended by Gabon’s NMCP, for their efficacy, tolerability, and safety for the treatment of uncomplicated *P. falciparum* malaria in Gabon. Previous studies performed in Lambaréné in 2000 reported similar PCR corrected cure rate of 94% for AS–AQ [26] and in 2009, 98.6% and 94.6% for AL and AS–AQ [8], respectively. In line with several other studies carried out in Africa, the results confirmed that AL and AS–AQ are still highly efficacious against *P. falciparum* [23–25]. However, a study performed 13 years ago (2004) in Lambaréné reported lower 28 day PCR corrected rate; of 86% for AS–AQ [27]; but this was an effectiveness study and crushed tablets of artesunate–amodiaquine were used, two important factors that may have influenced the treatment outcome.

The PCR-uncorrected efficacy was 78% and 89% for AL and AS–AQ, respectively. Relatively, these efficacies rates were low. However, similar uncorrected efficacies rates were reported in Angola, Ghana, and Nigeria and reflect the high level of ongoing malaria transmission in these regions [28–30]. The study was carried out during the high transmission period of malaria. Therefore, participants were continuously exposed to reinfection. This was confirmed by PCR analysis. Indeed, among the 15 cases of reappearing malaria parasites reported in the study, 10 were due to re-infection (7 for AL and 3 for AS–AQ).

There is no a strain of resistance to artemisinin in this study. Indeed, no mutation in *Pfkelch 13* associated with artemisinin resistance was identified. This observation was in accordance with previously published studies from Central Africa [9, 31] and worldwide mapping study [22].

The parasite clearance estimator was not calculated in this study. However, three participants presented with parasites on day 2 (2 for AL and 1 for AS–AQ). This is, in line with previous studies, suggesting that artemisinin-based combinations have a rapid effect on parasite clearance [32–34]. In the same order, fever clearance was fast in both groups. This confirmed the previous reports [35, 36]. Both drugs were well tolerated as reported in most studies [37–39], but adverse events were more frequent in the AS–AQ group as reported in others studies [40–42]. In most health facilities in Gabon, AL is prescribed at higher rates than AS–AQ because of patients’ complaints following the use of AS–AQ.

Regarding biological aspects, mean haemoglobin concentration observed at day 28 post-treatment was increased in both groups but was statistically significantly higher in the AS–AQ group and not in the AL group. There are equivocal results from previous studies on the effect of ACT on post-treatment haemoglobin levels [30, 43–45].

The main limitation of this study was that participants were not allocated randomly to the treatment arm. Therefore, a formal comparison between both groups was not performed. However, the therapeutic outcome of each artemisinin-based combination suggests that both remain efficacious to treat uncomplicated *P. falciparum* malaria in Lambaréné, Gabon.

## Conclusions

This study revealed that artemether–lumefantrine and artesunate–amodiaquine are safe, well tolerated and efficacious. They should remain the anti-malarial drugs of choice for the treatment of uncomplicated *P. falciparum* malaria in Lambaréné. Nevertheless, regular monitoring of the efficacy and gene mutation for resistance are required. Finally, it is important to conduct this study in other localities of the region to provide an overview of the efficacy of these first line artemisinin-based combinations at the country level.

## Supplementary information

**Additional file 1: Table S1.** Pfmsp1 genotyping by capillary electrophoresis. **Table S2.** Pfmsp2 genotyping by capillary electrophoresis.

## Data Availability

We have reported all the findings in the manuscript. The patient information sheet is available in institutional computer at Centre de Recherches Médicales de Lambaréné (CERMEL) This contains a unique identification code and patient personal details such as name, age, home address, etc. We cannot deposit the data (patients’ information sheet) in a public repository as the Institutional Ethics Committee does not permit this. If anyone wants to look at or use the data set, they should contact the corresponding author.

## Abbreviations

ACPR: adequate clinical and parasitological response
ACT: artemisinin‑based combination therapy
AL: artemether–lumefantrine
AS: artesunate
AQ: amodiaquine
CEI: Institutional Ethical Committee
CNE: The National Ethics Committee
CERMEL: Centre de Recherches Médicales de Lambaréné
ETF: early treatment failure
EDTA: ethylenediaminetetraacetic acid
LCF: late clinical failure
LPF: late parasitological failure
NMCP: National Malaria Control Programme
PCR: polymerase chain reaction
TBS: thick blood smear
WHO: World Health Organization
