# Supplementary material for: Monitoring of efficacy, tolerability and safety of artemether–lumefantrine and artesunate–amodiaquine for the treatment of uncomplicated Plasmodium falciparum malaria in Lambaréné, Gabon: an open-label clinical trial
Source: Malar J. 2019 Dec 16;18:424. doi: 10.1186/s12936-019-3015-4 (PMC6916217; doi:10.1186/s12936-019-3015-4)
Supplement: Supplementary file 1 — Additional file 1: Table S1. Pfmsp1 genotyping by capillary electrophoresis. Table S2. Pfmsp2 genotyping by capillary electrophoresis. [file 12936_2019_3015_MOESM1_ESM.pdf]

Additional file

Table S1:Pfmsp1 genotyping by capillary electrophoresis

Day	VID	K1	RO33	MAD20	Interpretation
Day 0	TET 011	242	0	0	recrudescence
Day 28	TET 011	242	0	194	recrudescence
Day 0	TET 012	187	0	0	new infection
Day 21	TET 012	224	0	0	new infection
Day 0	TET 013	187	0	203	new infection
Day 28	TET 013	224	0	0	new infection
Day 0	TET 014	242	0	0	new infection
Day 28	TET 014	0	154	0	new infection
Day 0	TET 020	242	154	212	recrudescence
Day 28	TET 020	242	0	0	recrudescence
Day 0	TET 025	0	0	212	new infection
Day 28	TET 025	168	0	0	new infection
Day 0	TET 028	224	0	0	ND
Day 21	TET 028	0	0	0	ND
Day 0	TET 033	242	0	0	recrudescence
Day 14	TET 033	242	0	0	recrudescence
Day 0	TET 039	0	154	0	new infection
Day 21	TET 039	0	0	194	new infection
Day 0	TET 050	204	0	0	new infection
Day 28	TET 050	224	0	0	new infection
Day 0	TET 051	0	154	194	recrudescence
Day 28	TET 051	0	154	175	recrudescence
Day 0	TET 054	204	154	0	new infection
Day 21	TET 054	153	0	0	new infection
Day 0	TET 064	242	154	0	recrudescence
Day 14	TET 064	242	154	194	recrudescence
Day 0	TET 074	194	154	0	new infection
Day 28	TET 074	212	0	0	new infection
Day 0	TET 094	0	154	194	new infection
Day 28	TET 094	224	0	0	new infection

Table S2: Pfmsp2 genotyping by capillary electrophoresis

Day	VID	IC_3D7.allele1	IC_3D7.allele2	IC_3D7.allele3	IC_3D7.allele4	FC27.allele1	Interpretation
Day 0	TET 011	NA	NA	NA	NA	NA	NA
Day 28	TET 011	NA	NA	NA	NA	NA	NA
Day 0	TET 020	247	326	344	384	478	new infection
Day 28	TET 020	283	NA	NA	NA	0	new infection
Day 0	TET 033	643	NA	NA	NA	446	recrudescence
Day 14	TET 033	NA	NA	NA	NA	446	recrudescence
Day 0	TET 051	643	NA	NA	NA	446	recrudescence
Day 28	TET 051	346	NA	NA	NA	446	recrudescence
Day 0	TET 064	279	NA	NA	NA	405	recrudescence
Day 14	TET 064	279	NA	NA	NA	406	recrudescence
